# Comparison of ultrahigh- and low-pressure packing methods for preparation of 50 cm long capillary UHPLC columns

**DOI:** 10.1016/j.chroma.2026.467400

**Published:** 2026-09-04

**Authors:** Tate A. Hancock, Robert T. Kennedy

**Affiliations:** a Department of Chemistry, University of Michigan, Ann Arbor, MI, 48109, USA; b Department of Pharmacology, University of Michigan, Ann Arbor, MI, 48109, USA

**Keywords:** Packing pressure, Capillary, Column efficiency, UHPLC, LC-MS

## Abstract

Interdependent variables including pressure, slurry concentration, and slurry solvent contribute to producing high-efficiency liquid chromatography columns by slurry packing. Most commonly, ultrahigh-pressure (over 2000 bar) slurry packing methods have been observed to generate the highest performing column packed beds. However, low-pressure packing (<200 bar) methods are also commonly used but have not been examined in depth or compared to ultrahigh-pressure packed columns for kinetic performance. In this work, we compare methods that employed low- (100 bar) or ultrahigh-pressures (2070 bar) to slurry pack 50 cm long × 75 μm inner diameter (i.d.) capillaries with 1.7 μm diameter ethylene-bridged hybrid (BEH) C18 particles. Both packing methods utilized a high slurry concentration, with low-pressure packing using a settled slurry (3 mm tall layer of particles in the bottom of a tube with slow stirring) and ultrahigh-pressure packing using a suspended (fast stirring to prevent settling) 200 mg/mL slurry. Columns were evaluated with isocratic separations of small molecules and gradient separations of a peptide standard mixture. Low-pressure packed columns saw an improvement in performance when sonication was used during packing. Low-pressure packed columns achieved a minimum reduced plate height as low as 1.18 and a peak capacity of 534 in <2 h, thus generating comparable separation performance to columns packed at ultrahigh-pressures. Similar column-to-column and run-to-run performance repeatability was also observed for both packing methods. The low-pressure packing technique provides a less technically demanding and more cost-effective approach for preparing high-efficiency capillary columns.

## 1. Introduction

Recent advancements in ultrahigh-pressure liquid chromatography (UHPLC) have focused on the implementation of sub-2.0 μm particles, which enable increased separation efficiencies and shorter analysis times [1]. To fully realize the theoretical gain in chromatographic performance from smaller packing materials, great care must be taken to ensure suitable column packing conditions. For this reason, systematic investigations have been performed exploring the column packing parameters that can increase packed bed homogeneity and lead to higher observed efficiencies [2,3]. In this work we compare low-pressure packing to the more commonly used ultrahigh-pressure methods for producing long, high-efficiency capillary columns.

Increasing separation efficiencies continues to play a pivotal role in advancing studies of biological systems. Combining sub-2.0 μm particles with relatively long columns (30–75 cm length) generates high separation efficiency, which has allowed for increased sample characterization for complex mixture analysis [4,5]. The advantages of miniaturization of column inner diameter (i.d.), including increased sensitivity with low flow nanoelectrospray ionization (nESI) coupled to mass spectrometry (MS), reduced mobile phase consumption, and compatibility with decreased sample volumes, have led many researchers to use columns with i.d. ≤ 100 μm (commonly called nano LC). Due to lower cost compared to commercially available nano LC columns and the option to customize column dimensions and packing material, many labs have opted to develop methods for preparing long nano LC columns in-house. Such columns have been used for high resolution separations of complex samples in fields such as proteomics, metabolomics, and lipidomics [6–11].

In an effort to determine capillary column packing conditions that yield the best column performance, previous works have investigated varying column i.d., slurry solvent, slurry concentration, packing rate, and implementation of sonication during packing [12–17]. Additionally, while many studies have focused on variations of a pressure-based slurry-packing method, alternative approaches such as centripetal force packing, slurry droplet packing, supercritical CO_2_ packing, electrokinetic packing, or techniques that focus primarily on mass fabrication of multiple columns at once have been explored [18,19]. Nevertheless, pressure-based slurry packing methods remain the most investigated and commonly employed for producing long, high-efficiency capillary columns. Tuning packing parameters is important as different conditions have been shown to result in more homogeneous column packed beds by reduction of geometrical and frictional wall effects, particle size segregation, and packed bed voids [20–22]. However, many of these packing parameters are highly interdependent, such as slurry concentration, packing pressure, and packing rate/time.

Most studies have used packing pressures of 1400–4100 bar to generate long, high-efficiency capillary columns [23–26]. However, previous studies are conflicting on the effects of varying packing pressure, with reports demonstrating an increase or no change in separation performance when packing pressure was increased in the ranges of 100–400, 690–2000, or 1000–3000 bar [6,27–29]. Presently, the highest reported efficiency for a long capillary column was achieved when packing with a combination of a high slurry concentration, sonication, and an ultrahigh packing pressure (2070 bar). Under these conditions, a minimum reduced plate height (h_min_) of 1.05 was reported for a 75 μm i. d. × 1 m long column packed with 1.9 μm ethylene-bridged hybrid (BEH) C18 [17].

One study reported using a pressure of only 100 bar for packing 75 μm i.d. × 50 cm long columns with sub-2 μm particles [30]. This method used a settled slurry layer for packing. Columns packed using low-pressure packing conditions yielded peak capacities that were comparable to those from commercially available columns of similar dimensions for the separation of a HeLa cell tryptic digest. It was suggested that by using a slowly rotating stir bar in contact with the column blank inlet, any clog formation due to high slurry concentration could be prevented. This clog prevention measure allows for continuous packing with the use of 100 bar to produce 50 cm long columns with sub-2 μm particles in <1 h. In contrast, the increased pressure and packing rate when using ultrahigh-pressures likely prevents similar clog formations. Using a low-pressure packing technique is of interest because it is less expensive and less technically demanding to perform compared to ultrahigh-pressure packing. In addition, previous work has suggested that lower packing pressures (amenable to a constant flow packing methodology) coupled with high slurry concentrations could be used to overcome packed bed axial heterogeneity inherent to variation in packing rates with constant pressure slurry packing [15,31]. To date, no works have investigated the difference in separation performance resulting from packing conditions that both utilize highly concentrated slurries, but varying packing pressures.

Here, we compare ultrahigh- and low-pressure packing conditions for the fabrication of 50 cm long capillary columns packed with 1.7 μm BEH C18 particles. Both packing techniques used concentrated slurries and a constant packing pressure. We determined that with a packing pressure of only 100 bar, capillary columns can yield an h_min_ as low as 1.18, thus achieving the same maximum performance demonstrated by columns packed at 2070 bar. Similarly, columns packed under either pressure demonstrated high peak capacities for separations of a peptide mixture.

## 2. Materials and methods

### 2.1. Chemicals and materials

L-ascorbic acid, hydroquinone, acetanilide, acetophenone, propiophenone, fluorescein, trifluoroacetic acid (TFA), acetone (HPLC Grade), acetonitrile (LC-MS Grade), and Whatman^®^ glass microfiber grade GF/C filter discs (1.2 μm pore size) were all purchased from Sigma-Alrich (St. Louis, MO). Formic acid (LC-MS Grade), formamide, hexanes, isopropanol (HPLC Grade), and 200 proof ethanol were all purchased from Fisher Scientific (Pittsburg, PA). HPLC grade 18 mΩ water was obtained from a Barnstead E-Pure system. Kasil 1 and Kasil 1624 were acquired from PQ Corporation (Malvern, PA). A Corning Costar 96-well plate was purchased from Fisher Scientific (Pittsburg, PA). Polyimide-coated fused-silica capillary with a 360 μm outer diameter (o.d.) and 20 or 75 μm i.d. were purchased from Polymicro Technologies, Inc. (Phoenix, AZ). 1.7 μm BEH C18 particles were donated by Waters Corporation (Milford, MA). All capillary tubing was polished using a capillary polishing station from ESI Source Solutions (Woburn, MA).

### 2.2. Frit preparation

For isocratic separations, UV absorbance detection occurred on-column 5 mm upstream of the outlet frit, where a section of polyimide coating had been removed by using a butane lighter. This approach allowed for column performance to be measured excluding extra-column effects due to the outlet frit or extra-column tubing. Frits were prepared at the outlet by modifying a previously reported procedure [23]. Briefly, a 2 cm window was burned 15 cm from the outlet end of the column blank. A fritting solution was made by combining Kasil 1624, Kasil 1, formamide, and 7.5 mM fluorescein solution (9:3:3:1) which was then vortexed and centrifuged for 5 min at 13,400 rpm. The outlet ends of column blanks were submerged into the fritting solution supernatant. A UV flashlight was used to monitor the fritting solution as it was drawn into the fused silica by capillary action. Once fritting solution partially filled the exposed window, the column blank was removed from the solution and a soldering iron set to 200 °C was touched directly above the fritting solution meniscus for 1 s 3–5 times, resulting in polymerization of a frit approximately 2–3 mm in length. Newly formed frits were flushed with 5 capillary volumes of isopropanol to remove any unpolymerized fritting solution. Finally, 7 bar of nitrogen pressure were used to purge isopropanol from column blanks before they were placed in an oven at 90 °C overnight to finish polymerization.

For columns used for gradient separations, outlet frits were prepared using Kasil and filter paper [32]. This fritting method was used to allow for column outlets to be directly connected to fused silica nESI emitters while introducing minimal extra-column volume. Briefly, a fritting solution of 1:1 v/v solution of Kasil 1 and 25% formamide (diluted in 18 mΩ water) was dispensed onto a piece of filter paper. One end of the column blank was pressed and spun into the fritting solution spot on the filter paper. Outlet frits were polished after at least one hour of polymerization at 90 °C, then placed back into the oven to finish polymerization overnight. Frits formed by this method were ~200 μm in length.

Inlet frits for all columns were prepared using Kasil and filter paper; however, inlet frits were polymerized for one hour at 90 °C, polished, and then allowed to finish polymerization at room temperature overnight. It was found that using a piece of tape to remove a small length of packed bed at the inlet (< 100 μm) before frits were added allowed for larger, more stable frits to be formed without noticeably affecting separation performance.

### 2.3. Column packing

For all columns, the following conditions were kept constant between all packing methods. Acetone was used as the slurry solvent. Particle slurries were vortexed and sonicated at 37 and 80 kHz on sweep mode for 10 min at each frequency before being transferred to a packing vessel. Column blanks were prepared by cutting a length of capillary that included the length of the desired packed bed plus an additional ~30 cm. The extended column blank length allowed for the entire packed bed to be submerged in an ultrasonic bath (Elma, Singen am Hohentwiel, Germany) during packing. Before packing, the column blank inlets were cleaned by first dipping them in acetone and then using a vacuum pump to remove residual acetone along with any dust, particulates, or water present. If sonication was used during packing, the column blank was pressed through a piece of foam and placed in an ultrasonicator bath set to 80 kHz on sweep mode. Ice was periodically added to the ultrasonicator bath to keep the temperature between 25–30 °C. After packing, columns were flushed at a constant mobile phase composition of 50/50 water/acetonitrile.

The apparatus and conditions used for ultrahigh-pressure packing of capillary columns has been previously reported (see Fig. 1A) [17]. Briefly, a particle slurry was prepared by weighing ~100 mg of particles to achieve a final slurry concentration of 200 mg/mL in acetone. After vortexing and sonicating, the slurry was then transferred to a packing vessel compatible with pressures > 2070 bar. A small stir bar was placed in the packing vessel slurry reservoir and set to a high stir speed to keep the particles suspended. The slurry was displaced with acetone into the column blanks using a pneumatic amplifier pump capable of 3450 bar (Haskel, Burbank, CA) through 4140 bar compatible valves (High Pressure Equipment Co, Erie, PA). The section of column blank that included the desired packed bed length was placed into an ultrasonic bath, with 2 cm of the outlet section above the water. Once the first 2 cm of the capillary were packed at ~70 bar, the pressure was quickly ramped over the course of 30 s to 2070 bar. The column was allowed to pack at 2070 bar until 5 cm beyond the desired packed bed length was achieved. The column was then depressurized until the packing rate substantially slowed down, then allowed to continue depressurizing for 15 min. After packing, columns were attached to a separate Haskel pump where 3450 bar inlet pressure was applied for 40 min. System pressure was then decreased to 690 bar and 5 temporary inlet frits were added above the desired packed bed length as described before [17]. The packed capillary columns were stored overnight before they were cut at the desired length and an inlet frit was installed.

The apparatus and conditions used for low-pressure packing of capillary columns has been previously reported (see Fig. 1B) [30]. Briefly, a particle slurry was prepared by weighing out enough particles to form a 3 mm tall layer of settled slurry in a 1.5 mL Eppendorf tube (~25–30 mg). After vortexing and sonicating, the slurry was allowed to settle into a dense layer in the bottom of the vial. A 2 mm tall stir bar was placed in the slurry vial and set to a slow stir speed (< 100 rpm) to help prevent formation of clogs at the column blank inlet, while also not disrupting the settled slurry layer. The slurry vial was transferred to a packing vessel compatible with pressures up to 170 bar. Column blanks were fixed to the packing vessel, pressed into the bottom of the slurry vial, and then retracted 2 mm and secured in this position. The placement of the column blank was performed to ensure that the inlet was submerged near the middle of the settled slurry layer and was in contact with the stir bar. Columns were packed either without the use of sonication during packing, as described originally [30], or with sonication. Packing was performed at a constant pressure of 100 bar until at least 5 cm beyond the desired bed length was achieved. After packing, columns were flushed, temporary frits were added, and columns were stored overnight as described for ultrahigh-pressure packed columns. However, columns that did not undergo sonication during packing were dipped 5 times for 1 s in an ultrasonic bath during flushing as described in the original method [30].

### 2.4. Isocratic separations and UV detection

Capillary columns were assessed using an isocratic UHPLC system described previously [33]. Briefly, two pneumatic amplifier pumps with maximum outlet pressures of 1030 and 5170 bar were connected in series and provided pressure for injection and separation, respectively. The mobile phase consisted of 50/50 water/acetonitrile, with 0.1% v/v TFA. L-ascorbic acid was used as a dead time marker (t_0_). A sample mixture of 5 mM L-ascorbic acid, 5 mM hydroquinone, 5 mM acetanilide, 0.1% v/v acetophenone, and 0.1% v/v propiophenone diluted in the mobile phase was utilized to assess column performance. The lower pressure pneumatic amplifier pump was used to perform injections by applying ~70 bar for 5 s, pushing a small sample plug (~5–10 nL) onto the column. The injection loop valves were then opened to flush the remaining sample solution out of the loop and closed again before applying the separation pressure. This injection apparatus introduces negligible extra-column variance and has been used extensively for characterizing high-efficiency capillary columns [17,23,25,33–35]. Operating pressures from 275 to 2400 bar were used for van Deemter curves. Columns were positioned in a PerSeptive Biosystems UVIS 205 Detector with an on-capillary flow cell, such that the 1 mm slit width for detection aligned with an exposed portion of packed bed that was ~5 mm upstream of the outlet frit. A 20 μm i.d. × 15 cm long empty capillary was attached post-column to maintain a more stable chromatographic baseline. Detection was performed at 224 nm.

Retention times and theoretical plate counts (N) were determined using an iterative statistical moments (± 3σ) algorithm written in Igor Pro 6.0 (Wavemetrics, Inc., Lake Oswego, OR) [36]. For calculating reduced plate height (h) and reduced linear velocities (v) for van Deemter curves, the Sauter-mean particle diameter (d_p,SA_) of the stationary phase particles and analyte diffusion coefficients (D_m_) in bulk mobile phase were measured. Scanning electron microscopy (SEM) for particle sizing analysis was performed on a JEOL (Peabody, MA) JSM-7800FLV and data was processed using ImageJ (National Institute of Health, Bethesda, MD). The d_p,SA_ for 1.7 μm BEH C18 particles was 2.13 μm (n = 225) by SEM analysis (Fig. S1). The reduced velocity for hydroquinone was calculated using the pressure-dependent diffusion in the bulk mobile phase [37]. For all other analytes, diffusion in the bulk mobile phase was determined by diffusion-ordered spectroscopy (DOSY) nuclear magnetic resonance (NMR) spectroscopy using a Varian (Palo Alto, CA) Vnmrs 700 NMR. The diffusion coefficients in bulk mobile phase for acetanilide, acetophenone, and propiophenone were determined to be 9.34 × 10^–6^, 1.15 × 10^–5^, and 1.07 × 10^–5^ cm^2^/s, respectively. Regression fit to the van Deemter equation was performed in GraphPad (Boston, MA) Prism 10.

### 2.5. LC-MS operation

Gradient separations were performed using a custom-built UHPLC system described previously with some modifications (see Fig. S2) [8, 38]. A Waters M-Class nano LC was used to generate mobile phase gradients and perform sample injections which were stored in a capillary and then driven onto the column using a constant pressure pneumatic amplifier pump. Valves were used to isolate the two pumps from each other during their respective operations. Pre-column fluidics consisted of a 20 μm × 35 cm viper nano capillary (Thermo Scientific, 6041.5240) that connected the LC injection valve to a 1/16″, 150 μm bore Vici tee (T1) with a PEEK plug in one side. The PEEK plug was loosened during separations to prevent any pressure buildup from damaging the LC system. A 20 μm i.d. × 75 cm long capillary was connected to T1, placed through a freeze/thaw (F/T) valve, and then connected to a 1/32″, 150 μm bore Vici tee (T2). The column and capillary gradient storage spool (100 μm i.d. × 50 m) were connected to T2. The column outlet was connected to a 20 μm i.d. × 16.5 cm long transfer capillary, which allowed for the entire column to be placed in a Waters column oven while still connecting to the mass spectrometer. The transfer capillary was connected to a nESI emitter (20 μm i.d. with 10 μm orifice and 5.5 cm long) with a conductive coating (New Objective, Littleton, MA).

All MS data was collected using a Waters Xevo QTOF operated in MS1 full scan mode with a scan range of 100–1575 *m/z*. For separation windows ≤ 100 min a scan rate of 0.3 scans/s was used. For separation windows > 100 min, the scan rate was decreased to 0.6 scans/s. Mobile phase A was water with 0.1% v/v formic acid (FA) and mobile phase B was 80/20 acetonitrile/water, with 0.1% v/v FA. The column oven was set to 40°C. All injections were performed in full loop injection mode with a 1 μL sample loop. The lyophilized peptide standard (MassPREP Digestion Standard Mix 2, Waters Corporation, Milford, MA) was resuspended in 1 mL of water with 0.1% v/v FA. The methods for gradient loading, sampling loading, and automated start for data acquisition are shown in Fig. S1. All gradient separations were performed at a constant separation pressure of 1720 bar. Varying separation windows for gradients was achieved by changing the volume of the linear portion of the mobile phase gradient. The system operated at a constant pressure, therefore mobile phase gradients were made by changing the total volume of the linear portion of gradient (8–52% MPB). Mobile phase gradients are described by their gradient rate in percent change in MPB per linear gradient volume normalized to the column volume (Δ%MPB/(gradient volume/column volume)). Column volumes were calculated assuming an interparticle porosity (ε) of 0.4. Columns were equilibrated for 40 min or longer at initial mobile phase conditions before each analysis.

Peak capacity measurements were determined by measuring the full width at half maximum (FWHM) of 18 peptides that eluted throughout the separation space. Chromatographic peak FWHM measurements were made using Peak Analyzer in Origin 2022 (OriginLab, Northampton, MA). The 4σ peak width was calculated by multiplying FWHM by 1.7. Peak capacities were then calculated by dividing the separation window by the average 4σ peak width. The separation window was defined as the difference in elution time between the first and last peak observed in all chromatograms.

## 3. Results and discussion

### 3.1. Isocratic column evaluation

Average packing times (n = 6) for ultrahigh- and low-pressure packed columns for a 75 μm i.d. × 50 cm long column were 8 ± 1 and 22 ± 2 min, respectively. Packing times were unaffected by sonicating the column during packing. The van Deemter curves were collected for columns packed under different conditions to assess column performance. Isocartic separations included compounds hydroquinone (k = 0.2), acetanilide (k = 0.5), acetophenone (k = 1.3), and propiophenone (k = 2.4). Most discussion will focus on hydroquinone because it has been used under similar conditions in prior work [17,22,33,39] and, as a weakly retained analyte, its performance is more sensitive to small changes in packed bed structure. Also, we found that acetanilide, acetophenone, and propiophenone had pressure dependent retention factors showing a 6–13% increase in k across the pressure range tested (275–2400 bar) (Fig. S3). The h_min_ for hydroquinone for all columns was achieved at an operating pressure between 630–1020 bar.

#### 3.1.1. Modifying low-pressure packing conditions

For long term packed bed stability, it is generally recommended to flush columns at a pressure 2–3 times higher than the columns will be operated at [3]. In our experience, the pressure that columns are flushed at does little to affect separation performance, but instead allows the packed bed to better resist compression at elevated pressures. Additionally, once the inlet packed bed begins to compress, a gap between the packed bed and column inlet is formed causing weakly retained analytes that are sensitive to extra-column effects to show increased amounts of band broadening and decreased efficiencies, while analytes that are more strongly retained or experience a focusing effect at the inlet of column will show little change. Therefore, we flushed columns at 3450 bar, the highest pressure possible on this system, for 40 min to reduce the possibility of a void forming at the column inlet from applied separation pressures.

Low-pressure packed columns were either prepared by dipping in an ultrasonicator bath during flushing, following the original reported procedure [30], or with continuous sonication during packing, a method found to be useful in ultrahigh-pressure packing [17]. As shown in Fig. 2, sonication during packing yielded a noticeable decrease in h_min_ in the van Deemter curve. This result suggests that sonication during packing improved packed bed homogeneity compared to only dipping in an ultrasonicator bath during flushing. This observation aligns with the previously demonstrated result that pairing a high slurry concentration with sonication during packing yields high-efficiency columns [17]. Sonication is likely disrupting the voids expected to be formed during packing due to the use of an agglomerating slurry solvent and a concentrated settled slurry, thereby decreasing column heterogeneity caused packed bed interstitial voids. As retention factor increases across the test compounds, a smaller reduction in the h_min_ is observed when sonication is utilized during packing (Fig. 2). Interestingly, even in the absence of sonication during packing, the low-pressure packed column achieved an h_min_ of 1.50 (313,100 plates/m). This result matches the h_min_ values achieved for similar long capillary columns packed at ultrahigh-pressures with a high slurry concentration and without the use of sonication [14,40]. Moving forward, sonication was implemented for all other low-pressure packed columns studied because of the lower h throughout the tested range of v.

#### 3.1.2. Comparing ultrahigh- and low-pressure packing

As shown in Fig. 3A, van Deemter curves of 2 replicate columns show little difference in their h_min_ whether packed using 100 or 2070 bar for hydroquinone. Replicate columns (n = 2) yielded h_min_ (hydroquinone) of 1.24 and 1.28 for ultrahigh-pressure packed columns and 1.18 and 1.30 for low-pressure packed columns. As a result, the maximum separation efficiency yielded by the packing methods was comparable, at 193,000 and 202,000 theoretical plates for ultrahigh- and low-pressure packed columns, respectively. This result demonstrates that high packing pressures are not required for producing high-efficiency packed columns. Instead, low-pressure with a high slurry concentration and sonication can be utilized. This high performance relies on the prevention of clog formation at the column inlet during the packing process through slow stirring. Furthermore, we have demonstrated that the implementation of sonication is useful when using a high slurry concentration during low-pressure packing to achieve the maximum separation performance. However, van Deemter curves for low-pressure packed columns had a steeper slope beyond h_min_, for hydroquinone, leading to worse performance at reduced linear velocities greater than 7 (pressures > 1380 bar). For example, at v = 9 (1780 bar separation pressure) h is 17% higher for 100 bar packed columns compared to those packed at 2070 bar.

For more retained species, comparable results are obtained across the velocity range tested (Fig. 3B, C, D). As the analytes’ retention factors increase, the difference in their slopes beyond h_min_ measured on different columns decreases. The smaller differences between packing conditions are due to analytes with greater retention factors being less impacted by small changes in the packed bed homogeneity when compared to weakly retained analytes. For propiophenone, h_min_ was observed at a separation pressure of ~1720 bar for all columns. Replicate columns (n = 2) produced h_min_ (propiophenone) of 1.66 and 1.74 for ultrahigh-pressure packed columns and 1.65 and 1.82 for low-pressure packed column replicates.

Previous works have demonstrated that decreasing the size of interstitial packed bed voids leads to increased observed linear velocities and column permeability [17,41]. As shown in Fig. 4, we observed no difference in linear velocity as a function of pressure between the column types. These results suggest that the size of packing voids and overall column permeability is similar for both packing methods. Therefore, a difference in interstitial void size is likely not a strong contributor to the increased slope in the van Deemter curves beyond the h_min_ seen in low-pressure packed columns. As discussed above, the use of sonication during packing likely played a role in limiting void size in both packing methods, leading to the observed trend in Fig. 4.

Although h_min_ values are similar, the low-pressure packed columns showed worse performance with higher flow rates for a weakly retained compound (hydroquinone). We hypothesize that this effect may relate to increased packed bed axial heterogeneity in columns packed at 100 bar. When packing at 100 bar, the initial 20 cm of packed bed formed within a few minutes, whereas the next 20 cm took roughly 20 min to pack. The difference in packing rates is likely due to the increasing back pressure generated as the packed bed forms and consolidates. Alternatively, ultrahigh-pressure packing allowed for the same latter section of the column packed bed to be formed in only 4 min, as well as a less obvious difference in packing rates experienced at the column inlet compared to the outlet. While gradients in packing rate are expected in constant pressure packing methods, the magnitude of the change in packing rate will be dictated by the slurry concentration, packed bed length, and packing pressure. Previous works have established that variations in packing rate impart column axial heterogeneity that can be characterized by increasing transcolumn heterogeneity (wall effects) when moving from a column’s outlet (most homogenous bed) to the inlet [15, 31]. Transcolumn heterogeneities caused by packing defects at the column wall can lead to increased band broadening via increased transcolumn eddy dispersion. This effect is especially true at higher linear velocities where transcolumn eddy dispersion can account for up to 70% of small molecule dispersion in long capillary columns [42]. Therefore, we propose that increased axial heterogeneity in low-pressure packed columns when moving from the column outlet to inlet is the source of the steeper slope beyond h_min_ [43]. Further limiting axial packed bed heterogeneity may be possible by maintaining an appropriate packing rate across the entire column. Maintaining packing rate could be accomplished by implementing a constant flow packing method. A low-pressure packing method is particularly advantageous towards developing a constant flow packing method as a lower starting packing pressure will in turn require a lower maximum packing pressure to maintain a set flow rate (packing rate). Conversely, attempting to operate under a constant flow for ultrahigh-pressure column packing would require pressures >> 2000 bar along with fittings, tubing, pumps, and a packing vessel that can be reliably operated at such high pressures, making constant flow packing unrealistic.

### 3.2. Gradient column evaluation using peptides

We further examined the difference in column performance by using gradient separations of a peptide mixture. For these tests, replicate 50 cm long × 75 μm i.d. columns packed with 1.7 μm particles using both methods were tested using different gradients with tryptically digested proteins as the sample. Separations were performed at 40 °C and a constant pressure of 1720 bar. The flow rate was 370–390 nL/min at a constant mobile phase composition of 52% MPB. The actual flow rate during separations varied throughout the gradient due to operating at constant pressure and changing mobile phase composition in the column. The separation pressure was selected to both further examine the performance difference observed at higher run pressures between packing methods and due to stable nESI spray being achieved reproducibly at this pressure. Representative base peak intensity (BPI) chromatograms are shown in Fig. 5 for a 0.5% gradient rate separation performed using an ultrahigh- or low-pressure packed column. Extracted ion chromatograms (EIC) of 18 selected peptides from the bottom trace are shown in Fig. 6. The 4σ peak width of these peaks was measured for every gradient separation for determining peak capacity. The distribution of peak widths observed for separations using a 0.5% gradient rate for each replicate column is shown in Fig. S5.

Across a wide range of separation windows, achieved by varying the gradient volume at the same pressure, columns packed at 2070 bar provided a moderately higher peak capacity when compared to 100 bar packed columns (Fig. 7). The maximum peak capacity yielded for a 190 min separation window by an ultrahigh-pressure packed column was 690, while the maximum peak capacity produced by a low-pressure packed column was 650 in a similar separation window. For separation windows > 10 min and < 100 min, ultrahigh-pressure packed generated an average of 21% higher peak capacities compared to low-pressure packed columns. For separation windows > 100 min, the difference in peak capacities measurements decreased with ultrahigh-pressure packed columns only producing an average of 10% higher peak capacities. The moderate increase in gradient separation performance under the described separation conditions aligns with the performance difference observed at high linear velocities for isocratic separations (Fig. 3A).

### 3.3. Column repeatability

For gradient separations, columns were tested over a period of at least one month and with 20 or more injections. Column performance and retention time repeatability were evaluated by performing injections in triplicate using a 0.5% gradient rate. The repeat separations were performed at different times during the columns’ use to assess possible signs of performance degradation. The first replicate separation was performed within the first 5 injections of a column’s lifetime, followed by two additional replicate separations performed as the last injections in a column’s use. A summary of column performance repeatability results is shown in Table 1. Columns packed under either method generate repeatable separation performance as shown by the small deviations in average peak widths and peak capacities. The average retention time relative standard deviation for 18 selected peptides for all columns was 1.1–4.6% (Table 1). These retention time deviations are less than those previously reported for peptide and derivatized metabolite separations performed used packed C18 capillary columns on the same custom UHPLC hardware [9,38]. Both column packing methods demonstrated good repeatability for peak capacity and retention time measurements. However, Table 1 also shows that slightly higher variation was observed between replicate separations on low-pressure packed column replicates.

## 4. Conclusions

We have presented results of modifying a low-pressure (100 bar) slurry packing method and compared it to a more thoroughly explored ultrahigh-pressure (2070 bar) packing method for preparation of 50 cm long capillary columns. The maximum separation efficiency achieved for the packing methods studied was 378,500 and 399,500 plates/m for ultrahigh- and low-pressure packed columns, respectively. Columns packed under either method achieved high peak capacities for peptides, with a maximum peak capacity of 690 and 650 achieved in roughly 3 h by an ultrahigh- and low-pressure packed column, respectively. As demonstrated in Fig. 3 and 4, replicate (n = 2) columns packed using either method had similar packed bed permeability and performance repeatability for isocratic separations, with a 9.7% difference between h_min_ achieved by low-pressure packed column replicates and a 3.2% difference for ultrahigh-pressure packed column replicates. Likewise, Fig. 7 and Table 1 illustrate that both packing methods give similarly repeatable peak capacities for gradient separations using replicate columns (n = 2) and for replicate separations (n = 3) performed throughout each column’s total use time. These results demonstrate that with only 100 bar of packing pressure, maximum separation performance and performance repeatability comparable to columns produced using 2070 bar can be obtained.

At mobile velocities above the optimal, low-pressure packed columns exhibited a steeper increase in h with v (mostly for low k analytes), leading to a 17% difference in h at high linear velocities. We hypothesize that differences in ultrahigh-pressure and low-pressure packed columns at high linear velocities arise from increased transcolumn heterogeneity moving from the column outlet to the inlet. Additionally, while one column dimension was investigated in this work (50 cm × 75 μm i.d.), it is known that changing column-to-particle diameter ratio (d_c_/d_p_) or column length can change column performance when packed using the same ultrahigh-pressure slurry packing method conditions [9,12,15,22,44]. Further studies will be required to determine if the reported low-pressure packing conditions also follow previously observed trends between column performance and column dimensions.

Low-pressure packing presents an alternative method for producing high-efficiency capillary columns that is less technically demanding, requires less expensive equipment, and utilizes ¼ of the packing material compared to the reported ultrahigh-pressure packing method. Furthermore, it is possible that implementing a constant flow packing method could improve axial homogeneity by reducing the variation in packing rates resulting from using a constant pressure packing technique. The use of such low pressures for preparing high-efficiency columns presents an interesting opportunity for implementation of a constant flow (constant packing rate) method as lower pressures will be needed to maintain a set packing rate.

## Supplementary Material

hancock si

Supplementary material associated with this article can be found, in the online version, at doi:10.1016/j.chroma.2026.467400.

## Figures and Tables

**Fig. 1.**
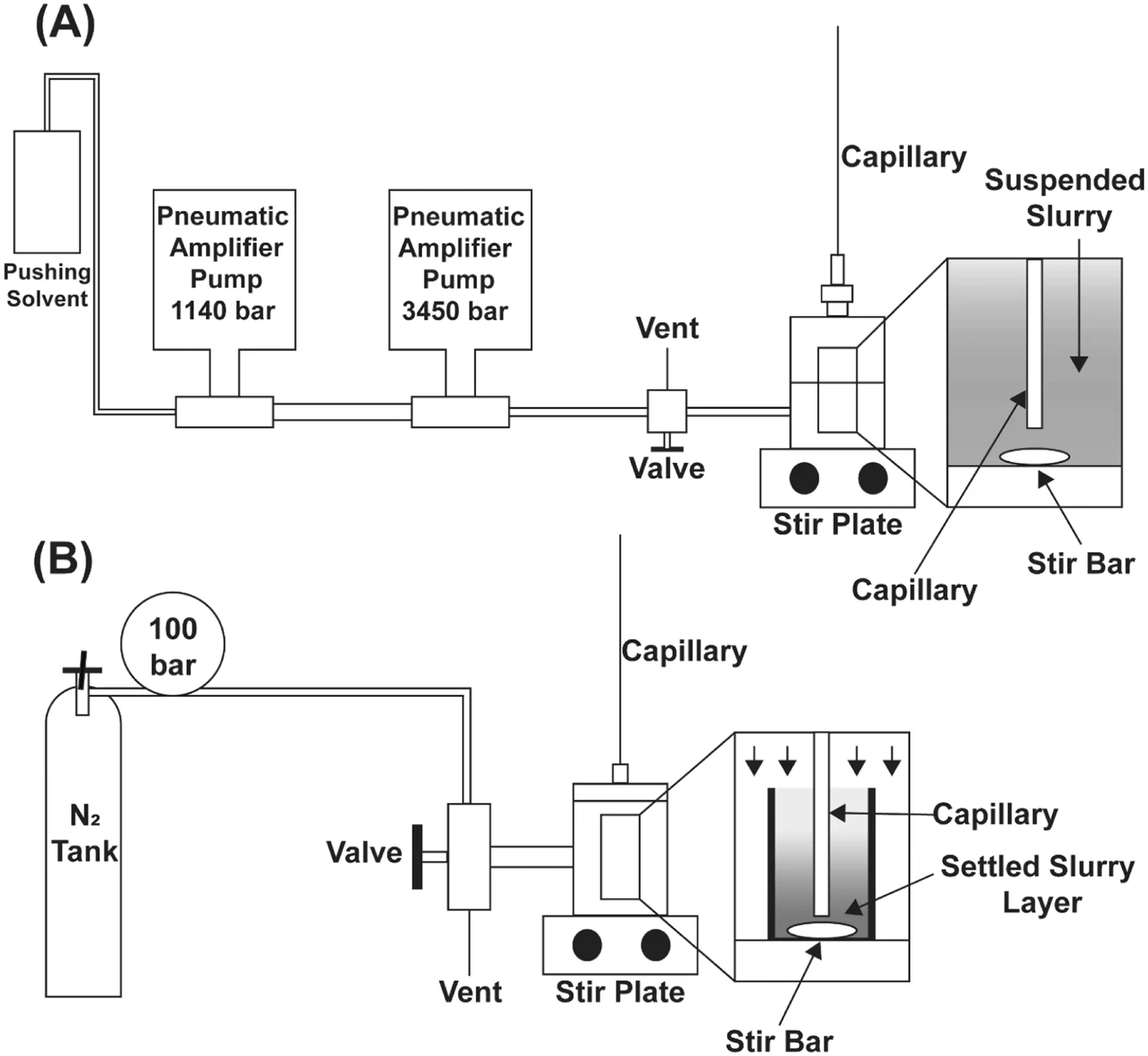
Schematic illustration of ultrahigh-pressure (A) and low-pressure (B) packing setups.

**Fig. 2.**
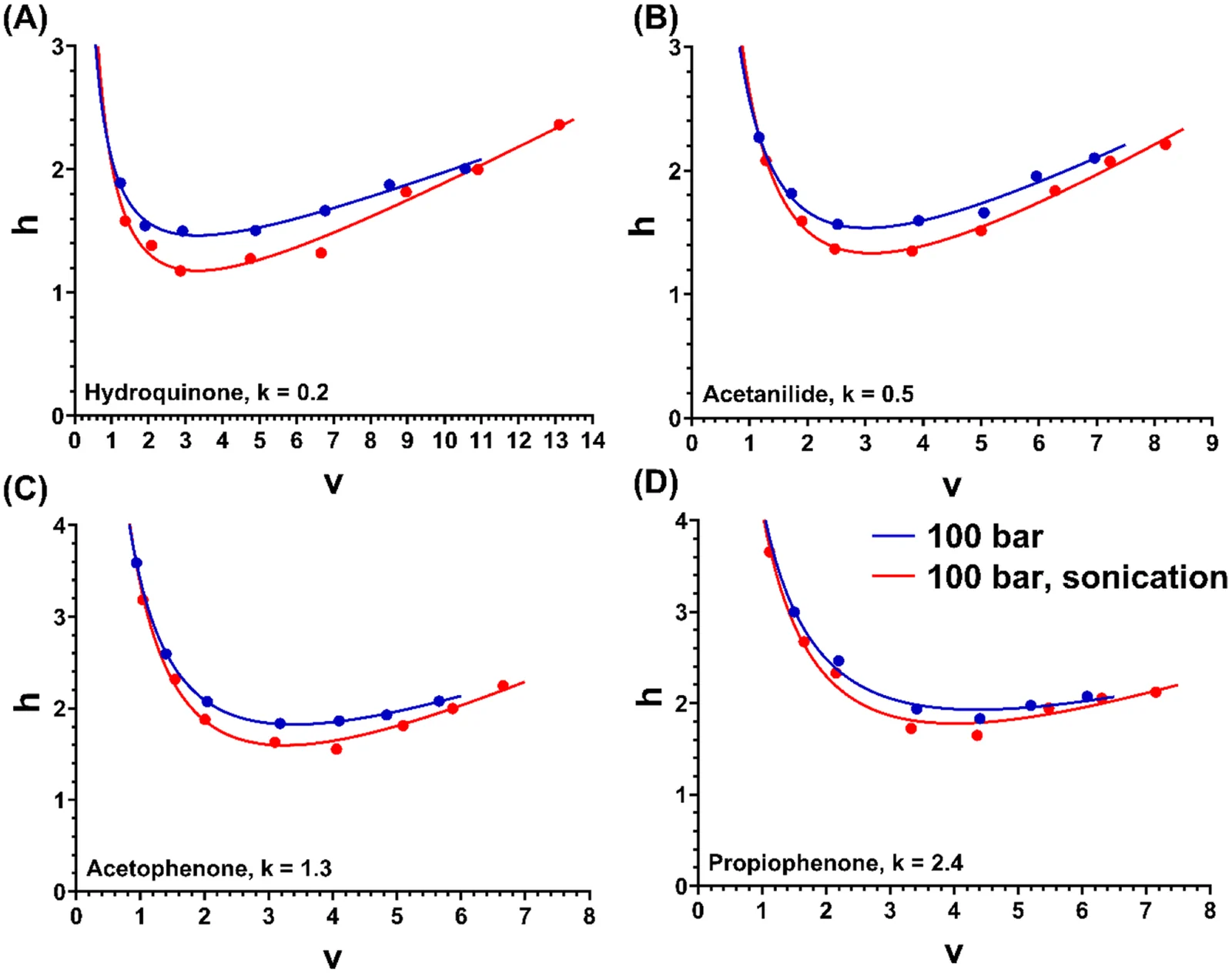
Plots of reduced van Deemter fits on columns packed at 100 bar with (red) or without (blue) sonication during packing. Plots for analytes hydroquinone (A), acetanilide (B), acetophenone (C) and propiophenone (D) are shown.

**Fig. 3.**
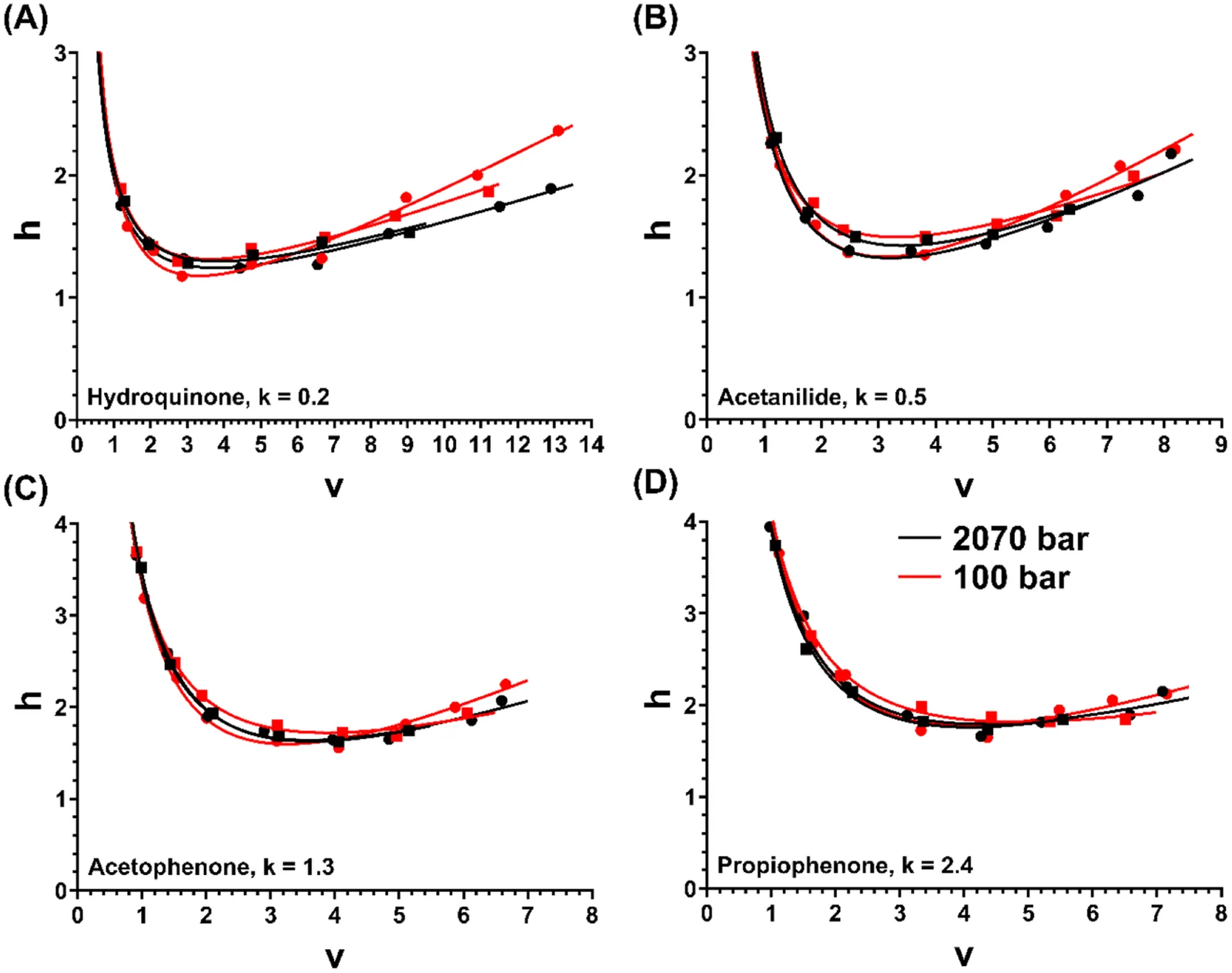
Plots of reduced van Deemter fits on columns packed using 2070 bar (black) or 100 bar (red). Plots for analytes hydroquinone (A), acetanilide (B), acetophenone (C) and propiophenone (D) are shown. Column replicates are designated by circles and squares.

**Fig. 4.**
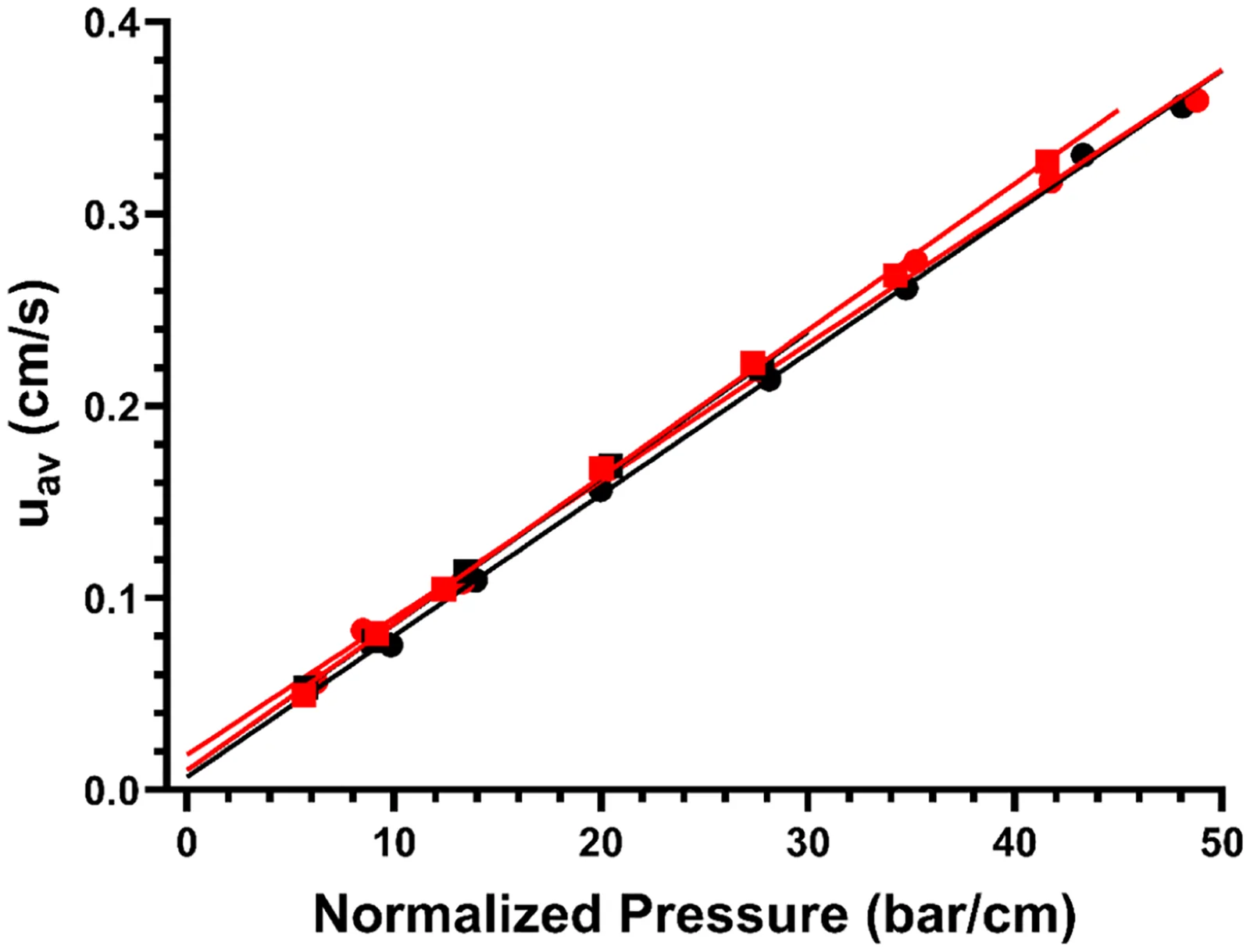
Plot of the linear velocity of the dead time marker, l-ascorbic acid, against pressure drop normalized for column length. Columns were packed at 2070 bar (black) or 100 bar (red). Column replicates are designated by circles and squares.

**Fig. 5.**
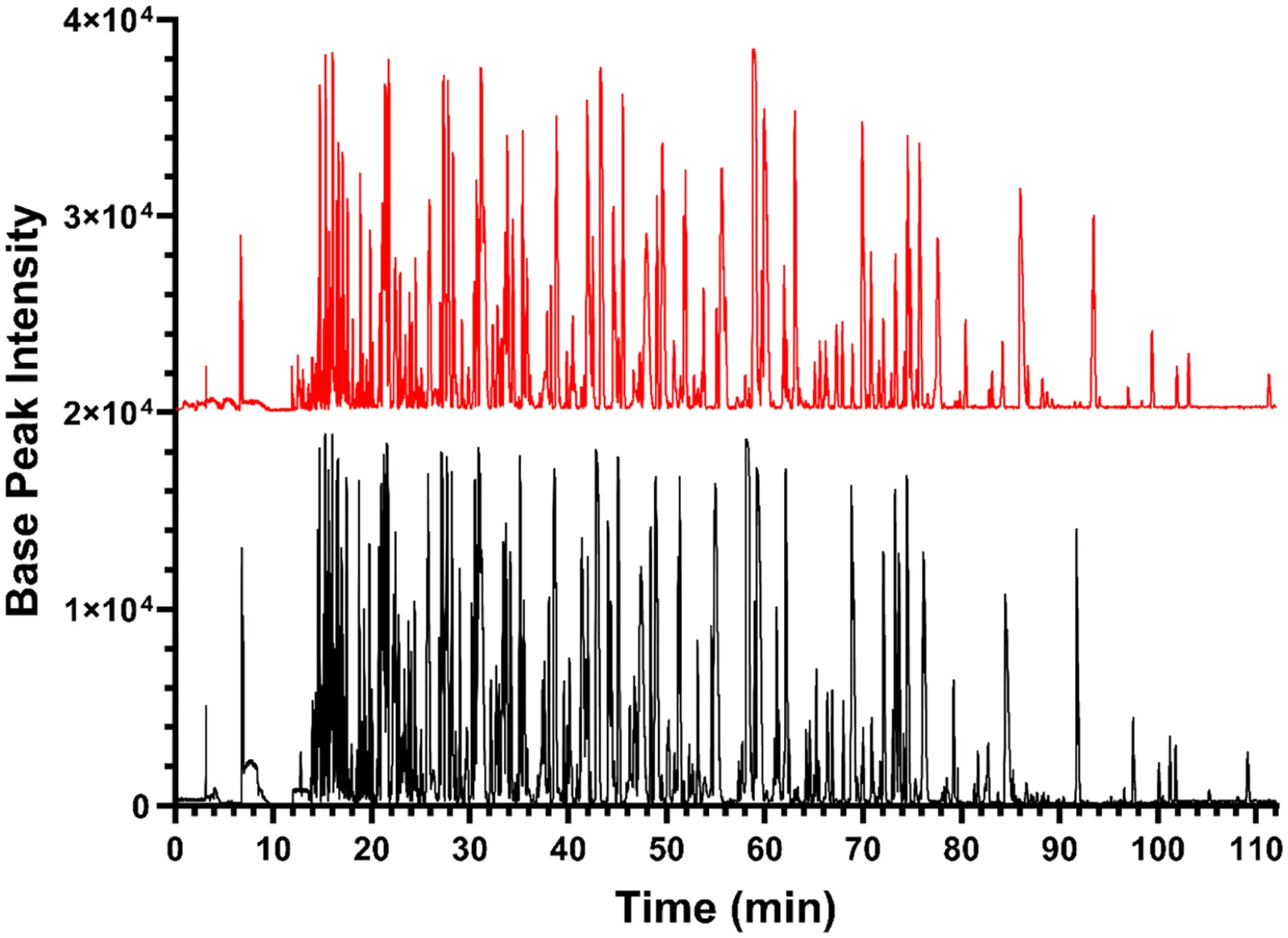
Representative gradient separation chromatograms of a tryptic digest on 50 cm × 75 μm i.d. × 1.7 μm d_p_ columns packed using either 2070 (black) or 100 bar (red). Gradient separations were performed using a 0.5% gradient rate for the gradient’s linear portion.

**Fig. 6.**
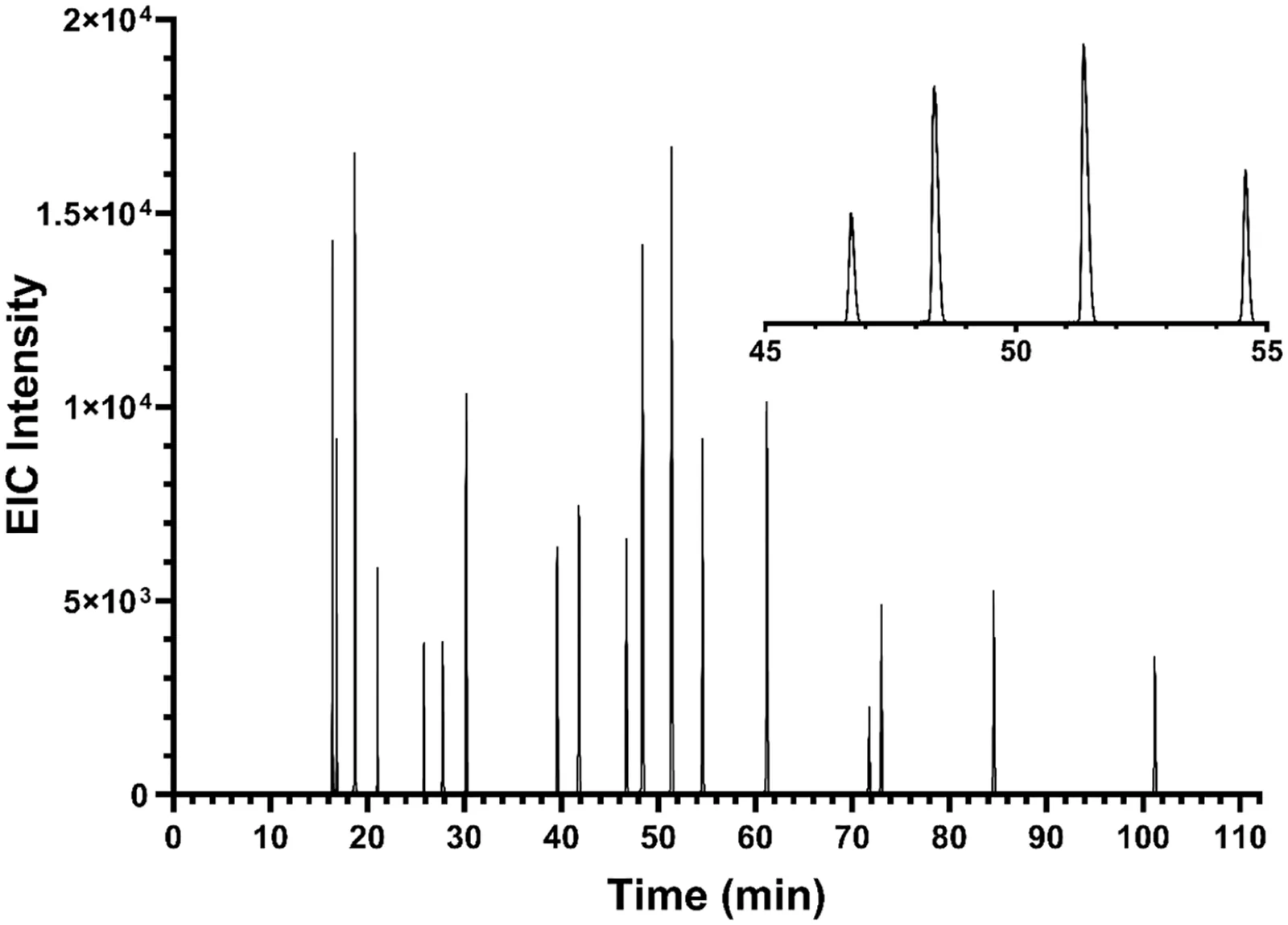
Extracted ion chromatograms of 18 selected peptide peaks from a tryptic digest. The extracted peaks shown are from the bottom chromatogram shown in Fig. 5. An inlayed 10 min section of the chromatogram is shown in the top right.

**Fig. 7.**
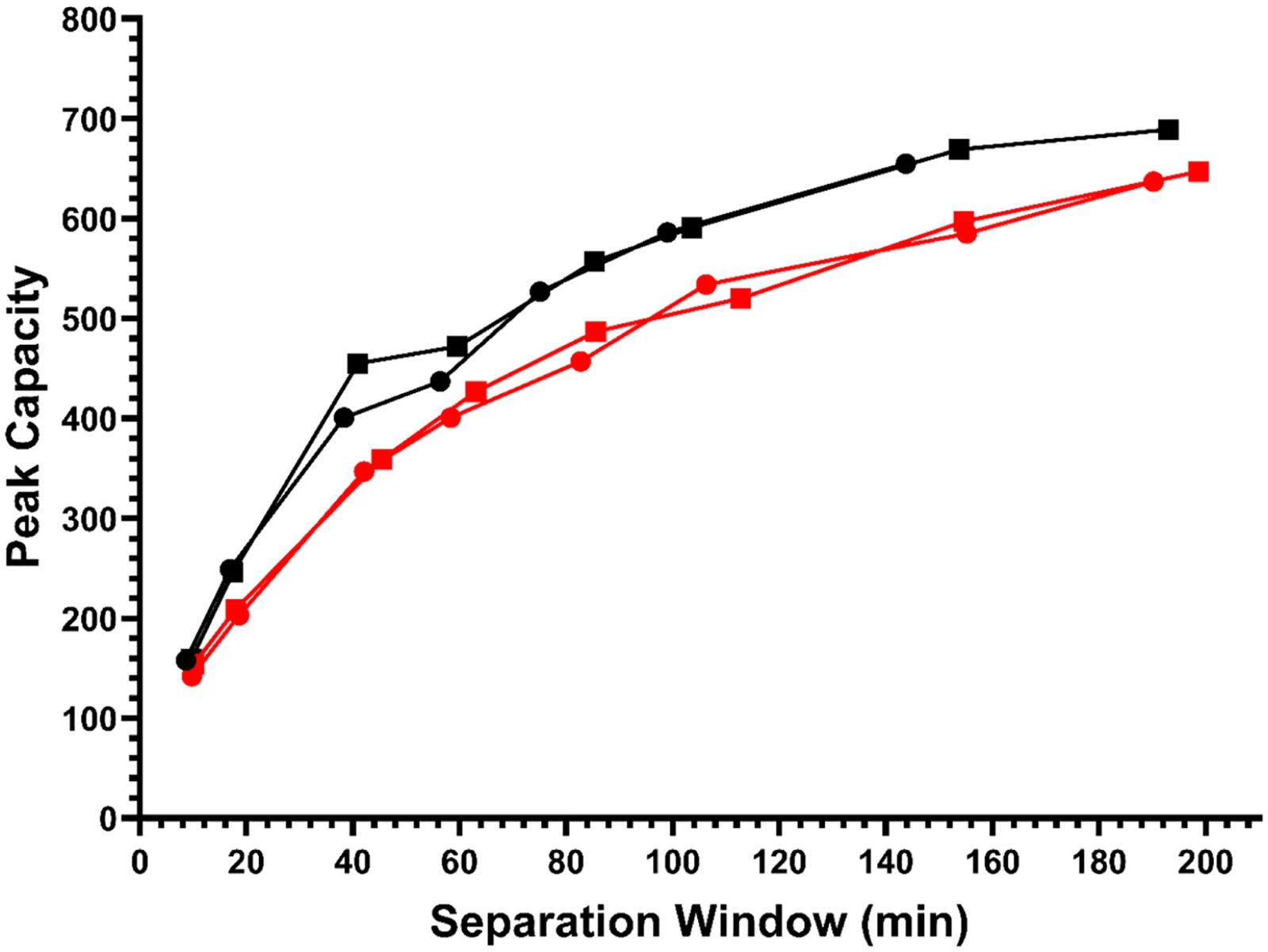
Peak capacity versus separation window for replicate columns packed using 2070 (black) or 100 bar (red). Replicate columns are denoted using circles and squares.

**Table 1:** Average and standard deviation for peak capacity and separation window and percent relative standard deviation (% RSD) of peptide retention times when performing replicate separations (n = 3) with a 0.5% gradient rate on column replicates packed at 2070 or 100 bar.

	AVG Peak Width 4σ (s)	Peak Capacity	Separation Window (min)	Retention Time % RSD
**2070 bar**				
Column 1	9.9 ± 0.3	590 ± 3	99 ± 3	2.2
Column 2	10.7 ± 0.2	590 ± 9	107 ± 3	2.2
**100 bar**				
Column 1	12.0 ± 0.3	530 ± 10	107 ± 1	1.1
Column 2	12 ± 1	550 ± 30	110 ± 4	4.6

## Data Availability

Data will be made available on request.
