# Supplementary material for: Comparison of ultrahigh- and low-pressure packing methods for preparation of 50 cm long capillary UHPLC columns
Source: J Chromatogr A. Author manuscript; Available in PMC 2026 Sep 30. (PMC13626020; doi:10.1016/j.chroma.2026.467400)
Supplement: Supplementary file 1 [file NIHMS2212299-supplement-hancock_si.docx]

***Supplemental Information for***

**Comparison of Ultrahigh- and Low-Pressure Packing Methods for Preparation of 50 cm Long Capillary UHPLC Columns**

Tate A. Hancock^1^, Robert T. Kennedy^1,2*^

1. Department of Chemistry, University of Michigan, Ann Arbor, MI 48109

2. Department of Pharmacology, University of Michigan, Ann Arbor, MI 48109

**1. Supplemental Figures** ……………………………………………………………………... 2-5

Figure S1. Stationary phase particle diameter measurement……………………………... 2

Figure S2. Custom-built UHPLC gradient system………………………………………... 3

Figure S3. Retention factor measurements……………………………………………….. 4

Figure S4. Gradient separation peak width distributions…………………………………. 5

**2. Supplemental Tables** ………………………………………………………………………. 6-7

Table S1. Sample and gradient loading………………………………………………....... 6

Table S2. Peptide retention time variation………………………………………………... 7

**3. Supplemental References** …………………………………………………………………..... 8

# ***1. Supplemental Figures***

**Figure S1.** Scanning electron microscopy (SEM) measurement of particle diameter (d_p_). SEM image of 1.7 µm BEH C18 particles Batch # 325 (B) and measured particle diameter distribution (A). The average d_p_ was 2.02 µm (indicated by the solid black line in the distribution) and the Sauter-mean diameter (d_p,SA_) was 2.13 µm (n = 225).

**Figure S2.** Schematic of the custom-built UHPLC system used for performing gradient separations. The system shown is a modified version of that reported in previous work.[1] T1 = 1/16”, 150 µm bore Vici tee, T2 = 1/32”, 150 µm bore Vici tee, T3 = 60 kpsi tee, High Pressure Equitpment, F/T valve = freeze/thaw valve.

**Figure S3.** Retention factor (k) versus separation pressure for analytes hydroquinone (circles), acetanilide (squares), acetophenone (triangles), and propiophenone (diamonds). A linear regression was fit to each data set.

**Figure S4.** Distribution of the peak widths at 4σ for 0.5% gradient rate separations performed on replicate columns packed at 2070 (A, B) or 100 bar (C, D).

# ***2. Supplemental Tables***

**Table S1.** Methods for gradient loading and sample loading, including automated switch on/off timing for freeze/thaw valve, pneumatic amplifier pump, and data acquisition (contact closure). The term “x” is modified to give differing gradient windows. Ex: x = 14.4 min for a 0.5% gradient rate for the linear portion of the gradient.

| Time (min) | Flow Rate (uL/min) | % MPB | Curve | Switch 1 (F/T Valve) | Switch 2 (Contact Closure) | Switch 3 (Pump) |
| --- | --- | --- | --- | --- | --- | --- |
| **Gradient Loading Method** | |  |  |  |  |  |
| Initial | 5 | 3 | - | off | off | off |
| 0.4 | 5 | 100 | 11 | off | off | off |
| 0.9 | 5 | 100 | 11 | off | off | off |
| 1.4 | 5 | 52 | 6 | off | off | off |
| x + 1.4 | 5 | 8 | 6 | off | off | off |
| x + 1.5 | 5 | 3 | 6 | off | off | off |
| x + 1.75 | 5 | 3 | 6 | off | off | off |
| x + 1.76 | 0.01 | 3 | 6 | off | off | off |
| **Sample Loading Method** | |  |  |  |  |  |
| Initial | 0.01 | 3 | - | off | off | off |
| 0.1 | 2.5 | 3 | 6 | off | off | off |
| 1.5 | 2.5 | 3 | 6 | off | off | off |
| 1.51 | 0.01 | 3 | 6 | off | off | off |
| 2.1 | 0.01 | 3 | 6 | **on** | off | off |
| 3.1 | 0.01 | 3 | 6 | on | off | **on** |
| 3.6 | 0.01 | 3 | 6 | on | **on** | on |
| 3.65 | 0.01 | 3 | 6 | on | **off** | on |

**Table S2.** Tracking retention time variation of 18 peptides with replicate (n=3) 0.5% gradient rate separations using columns packed at 2070 or 100 bar.

|  |  |  | **2070 bar** |  | **100 bar** |  |
| --- | --- | --- | --- | --- | --- | --- |
| **Peak Index** | **Peptide** | **m/z** | **Column 1 RT (min)** | **Column 2 RT (min)** | **Column 1 RT (min)** | **Column 2 RT (min)** |
| 1 | IETMR | 325.2 | 14.4 ± 0.3 | 16.6 ± 0.3 | 16.4 ± 0.2 | 17 ± 1 |
| 2 | ANIDVK | 330.2 | 14.9 ± 0.3 | 17 ± 0.3 | 16.8 ± 0.2 | 18 ± 1 |
| 3 | IATAIEK | 373.2 | 16.7 ± 0.4 | 19 ± 0.3 | 18.9 ± 0.2 | 20 ± 1 |
| 4 | VSALYK | 340.7 | 18.9 ± 0.3 | 21.4 ± 0.3 | 21.2 ± 0.2 | 22 ± 1 |
| 5 | GYNAQEYYDR | 639.8 | 23.1 ± 0.4 | 26.2 ± 0.4 | 25.9 ± 0.2 | 27 ± 1 |
| 6 | IPELR | 314.2 | 25.2 ± 0.5 | 28.3 ± 0.5 | 28.1 ± 0.2 | 29 ± 1 |
| 7 | VLGIDGGEGK | 472.8 | 27.5 ± 0.5 | 30.8 ± 0.6 | 30.6 ± 0.3 | 32 ± 1 |
| 8 | AWEVTVK | 416.7 | 36.1 ± 0.6 | 40.4 ± 0.8 | 40.1 ± 0.4 | 42 ± 2 |
| 9 | TNFDAFPDK | 527.7 | 38.3 ± 0.7 | 42.8 ± 0.9 | 42.5 ± 0.4 | 44 ± 2 |
| 10 | ANGTTVLVGMPAGAK | 693.9 | 43 ± 0.9 | 48 ± 1 | 47.6 ± 0.5 | 50 ± 2 |
| 11 | LVVSTQTALA | 501.8 | 44.8 ± 0.8 | 50 ± 1 | 49 ± 1 | 51 ± 2 |
| 12 | SISIVGSYVGNR | 626.3 | 47 ± 1 | 53 ± 1 | 52 ± 1 | 55 ± 2 |
| 13 | VFADYEEYVK | 631.8 | 50 ± 1 | 56 ± 1 | 56 ± 1 | 58 ± 2 |
| 14 | VIFLENYR | 527.3 | 56 ± 1 | 63 ± 1 | 62 ± 1 | 65 ± 3 |
| 15 | IGEEYISDLDQLR | 775.9 | 66 ± 2 | 74 ± 2 | 73 ± 1 | 76 ± 3 |
| 16 | LLSYVDDEAFIR | 720.9 | 68 ± 2 | 75 ± 2 | 75 ± 1 | 78 ± 3 |
| 17 | AVDDFLISLDGTANK | 789.9 | 79 ± 2 | 87 ± 3 | 87 ± 1 | 90 ± 3 |
| 18 | DAFLGSFLYEYSR | 784.4 | 94 ± 2 | 104 ± 3 | 104 ± 1 | 108 ± 4 |
|  |  |  |  |  |  |  |
|  |  | **AVG % RSD RT** | 2.2 | 2.2 | 1.1 | 4.6 |

# ***3. Supplemental References***

[1] K.M. Grinias, J.M. Godinho, E.G. Franklin, J.T. Stobaugh, J.W. Jorgenson, Development of a 45 kpsi Ultrahigh Pressure Liquid Chromatography Instrument for Gradient Separations of Peptides Using Long Microcapillary Columns and Sub-2 µm Particles, Journal of chromatography. A 1469 (2016) 60-67. https://doi.org/10.1016/j.chroma.2016.09.053.
